# Long-Term Course of Neural Autoantibody-Associated Psychiatric Disorders: Retrospective Data from a Specifically Immunopsychiatric Outpatient Clinic

**DOI:** 10.3390/antib12020034

**Published:** 2023-05-08

**Authors:** Niels Hansen, Kristin Rentzsch, Sina Hirschel, Claudia Bartels, Jens Wiltfang, Berend Malchow

**Affiliations:** 1 Department of Psychiatry and Psychotherapy, University Medical Center Göttingen, 37075 Göttingen, Germany; sina.hirschel@med.uni-goettingen.de (S.H.); claudia.bartels@med.uni-goettingen.de (C.B.); jens.wiltfang@med.uni-goettingen.de (J.W.); berend.malchow@med.uni-goettingen.de (B.M.); 2 Clinical Immunological Laboratory, 23627 Groß Grönau, Germany; k.rentzsch@euroimmun.de; 3 German Center for Neurodegenerative Diseases (DZNE), 37075 Göttingen, Germany; 4 Neurosciences and Signaling Group, Institute of Biomedicine (iBiMED), Department of Medical Sciences, University of Aveiro, 3810-193 Aveiro, Portugal

**Keywords:** neural autoantibody, psychiatry, outpatients

## Abstract

**Background:** Autoantibody-associated psychiatric disorders are a new terrain that is currently underrepresented considering immunopsychiatry’s potential importance for therapeutic aspects. The aim of our research was thus to present initial pilot data on the long-term clinical course of our patients in an outpatient clinic specializing in autoantibody-associated psychiatric disorders. **Methods:** Thirty-seven patients were examined clinically in our outpatient clinic at regular intervals over a 1.5-year period. We collected clinical data on their demographics, psychopathology, and cognition, and magnetic resonance imaging (MRI) and cerebrospinal fluid (CSF) data as well as the status of neural autoantibodies in blood and/or serum. **Results:** Our main finding was that affective, psychotic, and cognitive symptoms did not change significantly over the 1.5-year period, thus revealing no progression. We divided the entire cohort of autoantibody-positive patients (n = 32) into subgroups consisting of patients with dementia (n = 14), mild cognitive impairment (MCI) (n = 7), psychotic disorders (n = 6), and a CSF profile of Alzheimer’s disease (n = 6). Relying on established classification schemes, we identified the following percentages in our autoantibody-positive cohort: 28% with autoimmune encephalitis, 15% with autoimmune psychosis, and 63% with autoimmune psychiatric syndromes. **Discussion:** These initial pilot results suggest that autoantibody-associated diseases do not show a significantly progressive course in the long-term and are often characterized by impaired verbal memory recall when cognitive impairment progresses to dementia. These initial data need to be verified in larger cohorts. We believe that this pilot study underscores the importance of promoting such a specialized outpatient clinic to better characterize various aspects of autoantibody-mediated psychiatric disorders.

## 1. Introduction

Neural autoantibody-associated psychiatric disease is an increasing phenomenon in psychiatry affecting different psychiatric patient groups [1,2,3,4,5,6,7,8,9,10,11,12,13,14,15,16,17]. It is a significant phenomenon associated with approximately 23% of affective disorders [16], 17% of schizophrenic disorders [16], and 27% of disorders with cognitive dysfunction [13]. However, as healthy subjects also exhibit neural autoantibodies, autoantibodies alone do not demonstrate an autoimmunological cause of symptoms. Other criteria must be applied to assess an autoimmune origin of symptoms, such as those criteria proving an assumed cause of autoimmune encephalitis [18], autoimmune psychosis [19], or autoimmune dementia [20]. In Germany, the clinical care of patients revealing a potentially autoimmune basis is inadequate in psychiatry. There are very few outpatient clinics in psychiatric university hospitals treating such patients in Germany that also offer comprehensive medical consultation and diagnostics. We established an outpatient clinic for autoantibody psychiatric disorders in 2020 to care for precisely these patients presenting neural autoantibodies and psychiatric symptoms. The therapy of such patient groups has become an integral part of specialty care at neurology clinics, but not psychiatric clinics. The aim of this paper is to present our initial cross-sectional data and long-term observations in psychiatric patients with autoantibodies in a university psychiatric hospital.

## 2. Methods

In this study, we included a total of 37 individuals who were treated in our special outpatient clinic for autoantibody-associated psychiatric disorders between 2020 and 2022. This special outpatient clinic is one of the very first outpatient clinics in Germany focusing on autoantibody-associated psychiatric disorders. Referrals were mainly made by physicians from our Department of Psychiatry and Psychotherapy. In rare cases, referrals were made by resident physicians or physicians from other hospitals for second opinions. Criteria from the tenth edition of the International Classification of Diseases (ICD10) were applied to classify dementia, mild cognitive impairment, and other psychiatric disorder. The latest diagnostic guidelines were followed to diagnose Alzheimer’s disease (AD), considering both clinical [21] and laboratory factors [22]. If phosphorylated tau 181 protein (p-tau181) was elevated in cerebrospinal fluid (CSF) and the amyloid-ß 42/40 (Aß42/40) ratio was lower in CSF than standard laboratory values, the AD criterion was considered filled in the laboratory according to recent criteria [22]. This study was conducted in accordance with the Ethics Committee of the University Medical Center Göttingen and complied with the current version of the Declaration of Helsinki.

### 2.1. Outpatient Setting

We have established an outpatient clinic offering patients the opportunity to be examined by a team of two doctors on a weekly basis. We have created a homepage accessible to patients in order to pre-select patient characteristics: https://psychiatrie.umg.eu/patienten-besucher/ambulanzen/autoantikoerper-vermittelte-psychiatrische-erkrankungen (accessed on 12 February 2023). We drafted questions for potential patients at the initial contact to help determine for whom the treatment we provide is suitable (Table S1A,B).

### 2.2. Neural Autoantibodies

Most patients have already undergone lumbar puncture and neural autoantibody tests before referral. The autoantibody groups we assessed were: first, autoantibodies against membrane-surface antigens and second, autoantibodies against intracellular antigens. The autoantibodies we studied in detail were autoantibodies against membrane-surface antigens such as anti-α-amino-3-hydroxy-5-methyl-4-isoxazolepropionic acid receptor 1/2 (AMPAR1/2), -N-methyl-D-aspartate receptor (NMDAR), -leucin-rich glioma inactivated protein I1 (LGI1), -dipeptidyl-peptidase-like protein 6 (DPPX), -contactin-associated protein-2 (CASPR2), -metabotropic glutamate receptor type 1/5 (mGluR1/5), -gamma-aminobutyric acid A/B receptor (GABAA/BR), and myelin oligodendrocytic protein (MOG). We also assessed autoantibodies against intracellular targets such as antibodies against anti-glutamic acid decarboxylase 65 (GAD65), glial fibrillary acid protein (GFAP), -Rho GTPase activating protein 26 (ARHGAP26), -Purkinje cell protein carbonic anhydrase-related protein VIII (CARPVIII), -SOX1, -Ma2, -amphiphysin, -CV2, -Ri, -Yo, -HuD, -Titin, -Zic4, and Tr/DNER. BIOCHIP mosaics with brain tissue and recombinant cells were used to study immunoglobulin G (IgG) autoantibodies. Neural autoantibody detection methods have already been described in detail [13,23]. In short, standard immunofluorescence assays with separate cell-based assays were performed for Hu, Ri, Yo, Tr/DNER, Ma/Ta, GAD65, amphiphysin, aquaporin 4, MOG, NMDAR, AMPAR, GABABR, LGI1, CASPR2, IgLON5, Titin, Zic4, and DPPX with a cut-off value for autoantibody positivity of 1:10. We ran standard immunofluorescence assays for ANNA3 (autoantibody-positivity threshold of 1:10) and anti-myelin antibodies with an antibody positivity threshold of 1:100). In addition, immunofluorescence assays that have not been thoroughly assessed to date were used with separate cell-based assays for glycine receptors, adaptor-related protein complex 3 subunit beta 2 (AP3B2), neurofascin 186, CARPVIII, KCNA2, mGluR1, mGluR5, GABAAR, ARHGAP26, and flotilin 1/2 antibodies with an autoantibody-positivity threshold of 1:10. ITPR1, Homer 3, GFAP, neurochondrin, and neurexin3alpha were examined by cell-based assays and immunofluorescence assays with a cut-off threshold of 1:100 for autoantibody positivity. Nonspecific neuropil antibodies with unknown target antigen were scored as present if neuropil seroimmunoreactivity of rat hippocampal or cerebellar slices was present after incubating serum IgG. Autoantibody positivity in our study means that the autoantibody level measured was above the threshold for that autoantibody. It refers to either a membrane-bound or intracellular autoantibody, or both types of autoantibodies. The autoantibody-positivity criterion is met if at least one measured neural autoantibody type is above the threshold for antibody positivity. We also employed a semiquantitative scale to evaluate three different intensity levels ranging from mild to moderate to strong immunoreactive intensity in our patients’ CSF and blood samples. This autoantibody detection work was done in the Clinical Immunological Laboratory Prof. Stöcker, Groß Grönau. We also investigated other autoantibodies such as serum thyroglobulin (TG) (positive if >14 IU/mL) serum thyroid-peroxidase autoantibodies (TPO) (positive if >6 IU/mL) via an enzyme-linked immunosorbent assay (ELISA) from Roche (Roche, Basel, Switzerland) in our interdisciplinary laboratory at the University Medical Center Göttingen. Anti-TPO IgG was measured in CSF by ELISA without evaluated cut-off values by the Clinical Immunology Laboratory, Groß Grönau, Germany. Thus, the presence of anti-TPO autoantibodies in CSF had no diagnostic or clinical significance and could not be used to substantiate statements claiming clinical relevance for patients.

### 2.3. Neuropsychology and Psychopathology

In addition to lumbar punctures, extensive neuropsychological testing was usually performed in advance in our clinic, including a CERAD (Consortium to Establish a Registry for Alzheimer’s Disease) test battery. We assessed the main domains such as disorders of consciousness, orientation disorders, memory and attention disorders, formal thinking disorders, worries and compulsions, delusions, perceptual disorders, hallucinations, ego disturbances, disorders of the affect, drive and psychomotor activity disorders, circadian disorders, and other psychopathological disorders using the AMDP (Arbeitsgemeinschaft für Methodik und Dokumentation in der Psychiatrie) system. In each psychopathology domain, the AMDP’s individual items were scored as to whether they were present or absent in the patients. We scored between 0 and 1 whether (score = 1) or not (score = 0) a patient exhibited a given characteristic in each psychopathology domain.

### 2.4. Cerebrospinal Fluid Examination of the Patients

CSF obtained by lumbar puncture was analyzed in the Neurochemistry Laboratory of the Department of Neurology at the University Medical Center Göttingen. We determined phosphorylated tau protein 181 (p-tau181) (pathological: >61 pg/mL), total tau protein (t-tau) (pathological: >450 pg/mL), amyloid-ß 42 (Aß42) (<450 pg/mL), amyloid-ß 40 (Aß40), the ratio amyloid ß 42/40 (Aß42/40 × 10) (pathological: <0.5), neuron-specific enolase (NSE) (pathological: >30 ng/mL), and S100B (pathological: >2.7 µg/mL). In patients with reduced Aß42/40 ratios and elevated p-tau181 levels studied in the Neurochemistry Laboratory of the Department of Neurology, University Hospital Göttingen, we cross-validated the measurement of Aß42/40 and p-tau181 in another laboratory (Laboratory of Clinical Neurochemistry and Neurochemical Diagnosis of Dementia, Department of Psychiatry and Psychotherapy, University Hospital Erlangen) using other cut-off values: the Aß42/40 ratio was pathological if it was <0.05, and p-tau181 levels were pathological if they were >50 pg/mL. The cell count, percentage of lymphocytes and monocytes, content of immunoglobulin G (IgG), immunoglobulin A (IgA) and immunoglobulin M (IgM) in the CSF were also determined, as were the ratio of CSF/serum albumin, IgG, IgA, and IgM, presence of blood–brain barrier disruption, and intrathecal IgG synthesis.

### 2.5. Statistics

Sigma Plot (Version 11, Inpixion, Palo Alto, CA, USA) was used for graphic representation and Sigma Stat (Version 11, Inpixion, Palo Alto, CA, USA) for statistical analysis. If the data were normally distributed, a Student’s *t*-test was run, and if the data were not normally distributed, the Mann–Whitney test was used. For multiple testing, the Bonferroni correction was applied. To compare frequencies related to psychopathology, therapeutic response and type of therapy, comorbid conditions, and gender, Fisher’s exact test was performed. The *p*-value was considered significant when under 0.05.

## 3. Results

### 3.1. Basic Cohort Characteristics and Characterization of Clinical Cohorts

Of the 37 patients included, 32 showed neural autoantibodies and 5 patients had no neural autoantibodies. Our baseline cohort of autoantibody-positive patients had received various psychiatric diagnoses (Table 1).

Seventeen patients had autoantibodies in serum only, while 13 had autoantibodies in serum and CSF. Autoantibodies were only detected in the CSF of 2 patients. Five patients presented more than one autoantibody (Table 1). We formed four subgroups from the autoantibody-positive patients: (a) a cohort with CSF-based Alzheimer disease (the reduced ratio of Aß42/40 and elevated p-tau181 levels were confirmed via two different laboratories with different cut-off values, see methods) (n = 6), (b) a cohort with dementia (n = 14), (c) a cohort with MCI (n = 7), and (d) a cohort with psychotic disorders (n = 6) (Table 2).

**Figure 1:**
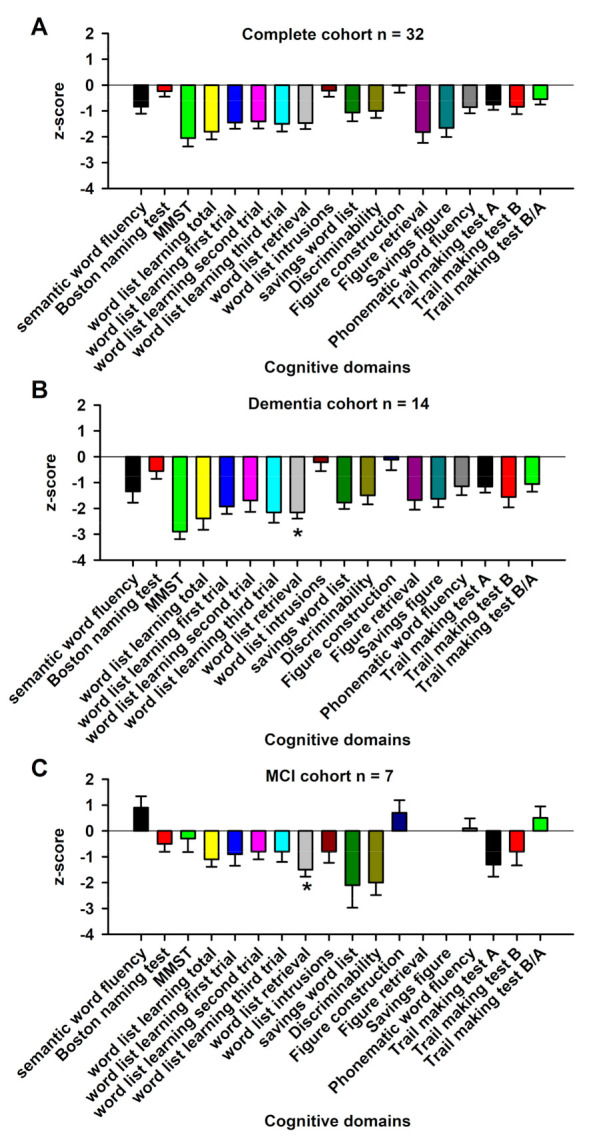
Cognitive profiles of the complete cohort, mild cognitive impairment patients, and dementia patients associated with neural autoantibodies. The cognitive profile, presented as z-scores of the CERAD test battery of the whole cohort, is shown in (**A**), dementia patients in (**B**), and mild cognitive impairment (MCI) patients in (**C**). Dementia patients with neuronal autoantibodies (**B**) were more impaired in verbal memory recall than mild cognitive impairment (MCI) patients with neuronal autoantibodies (**C**) * *p* < 0.005.

However, note that some patients, all with AD, also belonged to the dementia group. Nevertheless, five patients belonged to none of the four subgroups (affective organic disorder and NMDAR antibodies (n = 1), GFAP astrocytopathy associated with ego disturbances and visual snow phenomenon (n = 1), somatoform autonomic dysfunction associated with GAD65 autoantibodies (n = 1), depressive disorder associated with amphiphysin autoantibodies (n = 1), and IgLON5 autoimmune encephalitis (n = 1)). After applying the standard classifications for autoimmune encephalitis [18], autoimmune psychosis [19], and autoimmune-based psychiatric syndromes [2] in our cohort, we identified 28% (9/32) of patients with autoimmune encephalitis, 16% (5/32) with autoimmune psychosis, and 63% (20/32) of patients with autoimmune-based syndromes (Table 2). After applying the criteria for possible and definite autoimmune encephalitis according to Graus [18], 9 of 32 (28%) had possible and 4 of 32 (12.5%) had definitive autoimmune encephalitis. All these cohorts differed significantly in age at first presentation (Table 2). Memory recall was more impaired in neural autoantibody-positive dementia patients than in MCI patients with neural autoantibodies (Figure 1, *p* < 0.05). The other cognitive domains such as semantic and phonematic word fluency, verbal and figural learning, and memory functions, apart from memory recall and cognitive flexibility, did not differ significantly between autoantibody-positive dementia and MCI patients. The three subgroups (dementia, MCI, and psychotic disorders associated with neural autoantibodies) showed no differences in the number of patients receiving immunotherapy, steroids, or rituximab (Table 3). However, in patients with psychotic disorders, IVIGs were administered in 50%, whereas patients with dementia and neural autoantibodies were given no IVIGs (Table 3). The three groups (dementia, MCI, and psychotic disorders associated with neural autoantibodies) did not differ in their response to immunotherapy for affective, psychotic, or cognitive symptoms (Table 3).

### 3.2. Cerebrospinal Fluid Results of Cohorts

#### 3.2.1. Protein Markers

Dementia patients with autoantibodies (19%) revealed a non-significant trend after Bonferroni correction to higher percentage of monocytes among all leucocytes than MCI patients with autoantibodies (11%) (Table 1, *p* = 0.04, Bonferroni correction of *p*-level: <0.005). In contrast, the percentage of lymphocytes to all leucocytes was as a non-significant trend higher in MCI patients (88%) than in dementia patients (77%) (Table 2, *p* = 0.03, Bonferroni correction of *p*-level: <0.005). Interestingly, the IgM content in CSF was also as a non-significant trend higher in MCI patients (0.6 ± 0.4 mg/L) than in dementia patients (0.27 ± 0.22 mg/L) (Table 2, *p* = 0.04, Bonferroni correction *p*-level: <0.005).

#### 3.2.2. Neurodegeneration Markers

Total tau protein CSF levels were higher in AD (403.2 ± 182 pg/mL) than psychotic patients (110.3 ± 67) and in dementia patients (333.8 ± 163 pg/mL) than in psychotic patients or MCI patients (184.1 ± 109, Table 2, *p* < 0.05). However, total tau protein levels were not pathological as indicated by cut-off values in all groups. The p-tau181 level was significantly increased in AD patients with autoantibodies, well above the cut-off values (85 ± 48.9 pg/mL), compared to psychotic patients with autoantibodies (21.3 ± 13.6 pg/mL) (Table 3, *p* < 0.05). In addition, p-tau 181 levels were higher in MCI with neural autoantibodies (47.6 ± 25.6 pg/mL) than in psychotic patients with neural autoantibodies (21.3 ± 13.6) (Table 2), but they did not exceed cut-off values. CSF Aß42 levels were lower in dementia (992.4 ± 529.4 pg/mL) than in MCI patients (1321.5 ± 529 pg/mL) (Table 2), and the Aß42/40 ratio was lower in AD patients (0.5 ± 0.3 pg/mL) than in psychotic patients (1.2 ± 0.6 pg/mL); it remained below cut-off values in AD patients with neural autoantibodies (Table 3). We observed no relevant changes in CSF NSE and S100B levels between groups.

#### 3.2.3. Long-Term Time Course of Patients

Long-term follow-up of all patients revealed no significant psychopathological changes between the first and the last presentation with therapeutic interventions. In particular, affective, psychotic, or cognitive symptoms did not change in a relevant way between the first and fourth visit (after 8.3 ± 3.1 months) and between the first and sixth visit (after 17.0 ± 5.1 months, Table 4).

## 4. Discussion

Our results suggest that autoantibody-associated disorders follow a moderate time course involving no relevant deterioration of psychopathological features for up to 1.5 years after initial admission. Such a moderate clinical course over the long run is also evident in other studies of specific autoantibody-associated disorders regarding figural and verbal memory [24]. A fascinating observation that we made was the trend difference in excessive lymphocytes and reduced monocytes in MCI compared to dementia patients with neural autoantibodies. This trend finding may suggest that lymphocytes also play a relevant role in the initial appearance of neural autoantibodies, consistent with CD8+T cell-dominant immunopathology, as a study [25] and a case report [26] showed in neuropsychiatric disease with intracellular autoantibodies. We might speculate that lymphocytes predominate only at early stages of cognitive impairment associated with neural autoantibodies, and that the proportion of lymphocytes decreases when cognitive impairment progresses to dementia with neural autoantibodies, e.g., during the stage of chronic inflammation. In dementia with autoantibodies, monocytes might play an important role in CNS inflammation, as highlighted in a recent review [27] on the inflammasome in neurodegenerative disorders. Another interesting aspect is our trend observation that IgM was higher in MCI patients than in those with dementia, suggesting that an acute immune response involving IgM production is stronger in MCI patients with autoantibodies. We know from studies with NMDAR autoantibodies that IgM synthesis coincides with IgG, but this is less pronounced than IgG synthesis [28]. These patients’ less severe cognitive dysfunction may be associated with the onset of autoimmunity rather than a later stage of advanced cognitive dysfunction, i.e., dementia together with neural autoantibodies. Our study’s most important finding confirms the benign course of autoantibody-associated disorders in psychiatry. Even patients with dementia disorders or those with an AD biomarker profile do not differ significantly from patients with MCI or psychotic disorders in the long-term course in their affective, psychotic, or cognitive symptoms. These initial pilot results suggest that the course of autoantibody-associated disorders observed in an outpatient setting is benign. The prognosis and course of cognitive disorders associated with autoantibodies in particular are therefore likely to be rather unremarkable and do not appear to exhibit the usual progression common to neurodegenerative disorders. Only our group with an AD profile in their CSF also demonstrated marked neurodegeneration with increased p-tau 181 and a decreased Aß42/40 ratio. 

### Limitations

Our subgroups are hard to compare due to their low patient numbers. Such small patient groups prevent valid conclusions regarding the long-term outcome of patients with autoantibody-associated psychiatric disorders. Nevertheless, this pilot study offers an initial perspective on the quite encouraging prognosis of autoantibody-associated psychiatric disorders. The paucity of such data, and the rarity of such outpatient clinics in the field of psychiatric diseases set an example, and provide evidence of how fruitful it is to be able to observe such patients over the long term. This benign long-term course is attributable to the fact that these patients’ first presentation was already after their first inpatient stay, during which they may have suffered from an autoantibody-associated disorder at a severe or acute stage, as these can sometimes be even more severe than psychotic disorders with other causes (i.e., anti-thyroid antibodies and negative symptoms in early-onset psychosis) [29]. The strength of this case series is our patients’ longitudinal observation over a 1.5-year period. There is research evidence of other such approaches in patients with chronic psychiatric disorders, e.g., chronic schizophrenia with evidence of systemic lupus erythematosus with antibodies to nuclear antibodies or double-stranded DNA [30] or even in psychosis due to neuropsychiatric lupus erythematosus with cell-surface autoantibodies [31]. Another study limitation is that we could create no control group in our study, as this study was retrospective and no healthy subjects presented in our special outpatient clinic. We therefore cannot compare the temporal development of neuropsychiatric symptoms over the 1.5-year period with controls. Note also that gender is a potential confounding factor in our subcohorts (see Table 2).

## 5. Conclusions

Our results promisingly suggest that more clinical data should be collected to better treat and assess this disease entity. Surprisingly, the long-term cognitive outcome in patients with neural autoantibodies was better than expected, however, it does not represent the usual course observed in patients with such psychiatric syndromes and disorders under therapy. 

## Figures and Tables

**Table 1:** Description of basic cohort.

Patient	Abs Serum	Abs CSF	Diagnosis	AE	AP	APS
1	Homer 3	Homer 3	MCI	0	0	1
2	Glycine receptor	-	MCI	0	0	0
3	GAD65	-	MCI	0	0	1
4	Titin	Titin	Alzheimer’s dementia	0	0	0
5	GAD65	GAD65	Catatonic schizophrenia	1	1	1
6	GAD65	GAD65	Alzheimer´s dementia	0	0	0
7	Titin, Neuropil	Titin, Neuropil	Mixed dementia	0	0	1
8	Neuropil	Neuropil	Dementia	0	0	1
9	GAD65	GAD65	Mixed dementia, Bipolar disorder	0	0	1
10	CV2/CRMP5	CV2/CRMP5	Organic delusional disorder	1	1	1
11	CARPVIII	-	Mixed dementia	0	0	1
12	-	NMDAR	Organic delusional disorder	1	0	1
13	Glycine receptor	-	Alzheimer´s dementia	0	0	0
14	GFAP, LGI1	-	Astrocytopathia Other stimulant dependence	1	0	1
15	NMDAR	NMDAR	Organic affective disorder	1	1	1
16	IgLON5	-	Mixed dementia	0	0	1
17	GAD65	-	Somatoform autonomic disorder	0	0	0
18	Amphiphysin	Amphiphysin	Depressive disorder, MCI	0	0	0
19	Neuropil	-	Depressive disorder, MCI	0	0	0
20	IgLON5	IgLON5	Autoimmune encephalitis	1	0	1
21	AP3B2	-	Brain trauma	0	0	1
22	Neuropil	-	Mixed dementia Major depressive disorder	0	0	1
23	mGluR5	-	MCI, autoimmune encephalitis	1	0	1
24	TPO	TPO	Organic delusional disorder	0	0	0
25	TG, TPO	-	Organic delusional disorder	1	1	1
26	Neuropil	-	MCI Mild depressive disorder	0	0	0
27	-	Zic4, Yo	Alzheimer´s dementia	0	0	0
28	Yo, GAD65	Yo	Alzheimer´s dementia	0	0	0
29	KCNA2		Alzheimer´s dementia	0	0	0
30	Flotilin 1/2		Dementia	0	0	1
31	Amphiphysin		Dementia	0	0	1
32	Recoverin		Paranoid schizophrenia	0	1	1

Abbreviations: Abs = autoantibodies, AE = autoimmune encephalitis, AP3B2 = adaptor-related protein complex 3 subunit beta 2; AP = autoimmune psychosis, APS = autoimmune based psychiatric syndrome, CSF = cerebrospinal fluid, CV2/CRMP5 = collapsin response-mediator protein 5, GAD65 = glutamic acid decarboxylase 65, GFAP = glial fibrillary acid protein, KCNA2 = potassium voltage-gated channel subfamily A member 2, LGI1 = leucin-rich glioma 1 inactivated protein, mGluR5 = metabotropic glutamate receptor type 5, NMDAR = N-methyl D-aspartate receptor, TG = thyreoglobulin, TPO = thyroid peroxidase.

**Table 2:** Clinical and cerebrospinal fluid characterization of special outpatient cohort.

Parameter	A Complete Cohort (n = 32)	B Cohort with AD (n = 6)	C Cohort with Dementia (n = 14)	D Cohort with MCI (n = 7)	E Cohort with Psychotic Disorders (n = 6)
**Basic Demographic Data**					
Age in years	59.4 ± 19	79.0 ± 6.2	73.1 ± 10.8 ^#,+^	60.7 ± 6.8 ^#,$^	42.8 ± 11.2 ^$,+^
Duration of symptoms in years	2.8 ± 3.2	1.9 ± 0.74	2.9 ± 1.9	4.7 ± 6.0	1.6 ± 0.9
Gender female/all patients	17/32 (53%)	3/6 (50%)	8/14 (57%)	5/7 (71%)	3/6 (50%)
**Comorbid Diseases**					
Diabetes mellitus	4/32 (12.5%)	1/6 (17%)	3/14 (28%)	1/7 (14%)	0/6 (0%)
Cardiovascular disease	13/32 (41%)	4/6 (67%)	8/14 (57%)	3/7 (21%)	1/6 (17%)
Lung disease	1/32 (3%)	0/6 (0%)	1/14 (7%)	0/7 (0%)	0/6 (0%)
Rheumatologic disease	0/32 (0%)	0/6 (0%)	0/14 (0%)	0/7 (0%)	0/6 (0%)
**CSF**					
Cell count (<5 µL)	3.2 ± 8.4	0.5 ± 0.83	0.9 ± 1.6	3.6 ± 8.5	9.3 ± 16.6
Lymphocytes %	80.5 ± 34	70.5 ± 37.83	77.0 ± 37.4 ^#^	88.0 ± 33.8 ^#^	79.4 ± 34.9
Monocytes %	16.2 ± 11.5	23.0 ± 13.8	19.1 ± 11.7 ^#^	10.5 ± 6.5 ^#^	16.4 ± 15.3
Total protein content mg/L	412. 3 ± 157.8	392.5 ± 91.6	433.2 ± 161.0	416.3 ± 169.8	329.2 ± 132.0
Albumin content mg/L	289.8 ± 122.1	270.8 ± 72.1	299.5 ± 118.5	296.1 ± 141.8	224.2 ± 95.8
IgG mg/L	36.1 ± 26.8	28.6 ± 6.6	32.4 ± 13.7	39.5 ± 24.0	42.2 ± 54.8
IgA mg/L	4.1 ± 2.8	5.6 ± 3.6	4.9 ± 2.9 ^+^	4.2 ± 3.6	2.3 ± 1.6 ^+^
IgM mg/L	0.5 ± 0.6	0.2 ± 0.1	0.3 ± 0.22 ^#^	0.6 ± 0.4 ^#^	0.8 ± 1.1
Ratio CSF/serum albumin	6.8 ± 2.9	6.4 ± 1.6	7.1 ± 2.75	6.7 ± 2.8	5.3 ± 2.4
Ratio CSF/serum IgG	3.7 ± 2.5	2.8 ± 0.8	3.4 ± 1.43	3.5 ± 1.9	4.4 ± 5.2
Ratio CSF/serum IgA	1.7 ± 0.9	1.5 ± 0.5	1.8 ± 0.74	1.8 ± 1.1	1.3 ± 0.9
Ratio CSF/serum IgM	0.6 ± 0.6	0.4 ± 0.2	0.4 ± 0.29	0.6 ± 0.4	0.9 ± 1.4
Intrathecal IgG synthesis	4/32	1/6	2/14	0/7	1/6
Blood–brain barrier disturbances	7/32	0/6	2/14	1/7	1/6
Tau protein (>450 pg/mL)	254.2 ± 159.8	403.2 ± 181.9 *	333.8 ± 163.1 ^#,+^	184.1 ± 108.6 ^#^	110.3 ± 6 7.2 *^,+^
P-tau181 (>61 pg/mL)	56.2 ± 35.5	85 ± 48.9 *	71.5 ± 37.1	47.6 ± 25.6 ^$^	21.3 ± 13.6 *^,+,$^
Aß42 (<450 pg/mL)	1121.5 ± 610.3	652.4 ± 279.0	992.4 ± 529.4 ^#,+^	1321.5 ± 528.4 ^#^	1000.6 ± 564.1
Aß40 pg/ml	11,366.7 ± 6083.8	10,434.0 ± 7022.9	11,282.5 ± 5528	12,791.0 ± 6134.8	8713.3 ± 4937.4
Ratio Aß42/40 × 10 (<0.5)	1.2 ± 0.9	0.5 ± 0.3 *	1.2 ± 1.17	1.1 ± 0.53	1.2 ± 0.6 *
NSE (>30 ng/mL)	21.8 ± 11.2	25.4 ± 13.5	22.5 ± 13.0	18.6 ± 10.5	.
S100B (>2.7 µg/L)	3.8 ± 2.4	3.48 ± 2.2	2.8 ± 1.95	2 ± 1.16	.

Abbreviations: * *p* < 0.05 B vs. E Mann–Whitney U-test, ^#^ *p* < 0.05 C vs. D Mann–Whitney U-test, ^+^ *p* < 0.05 C vs. E Mann–Whitney U-test, ^$^ *p* < 0.05 D vs. E Mann–Whitney U-test. AD = Alzheimer´s disease, amyloid-ß 40 = Aß40, amyloid-ß 42 = Aß42, CSF = cerebrospinal fluid, IgA = immunoglobulin A, IgG = immunoglobulin G, IgM = immunoglobulin M, MCI = mild cognitive impairment, NSE = neuron-specific enolase, ratio amyloid Aß42/40 = ratio amyloid Aß42/40. Values are expressed as means.

**Table 3:** Time course of psychiatric patients cohorts with neural autoantibodies.

Parameter	A Cohort with Dementia (n = 14)	B Cohort with MCI (n = 7)	C Cohort with Psychotic Disorders (n = 6)
Immunotherapy	5/14 (36%)	2/7 (29%)	5/6 (83%)
Steroids	4/14 (29%)	2/7 (29%)	3/6 (50%)
IVIGs	0/14 ^+^ (0%)	0/7 (0%)	3/6 ^+^ (50%)
Rituximab	0/14 (0%)	0/7 (0%)	1/6 (17%)
**Affective Symptoms**			
Improvement	1/14 (7.1%)	1/7 (14%)	0/6 (0%)
Deterioration	1/14 (7.1%)	2/7 (29%)	1/6 (17%)
Stable	1/14 (7.1%)	2/7 (29%)	0/6 (0%)
**Psychotic Symptoms**			
Improvement	1/14 (7.1%)	1/7 (14%)	5/6 (83%)
Deterioration	0/14 (0%)	0/7 (0%)	0/6 (0%)
Stable	0/14 (0%)	0/7 (0%)	1/6 (17%)
**Cognitive Dysfunction**			
Improvement	2/14 (14%)	1/7 (14%)	0/6 (0%)
Deterioration	6/14 (43%)	4/7 (57%)	1/6 (17%)
Stable	6/14 (43%)	1/7 (14%)	4/6 (67%)

Abbreviation: IVIG = intravenous immunoglobulins, MCI = mild cognitive impairment. Fisher´s exact test was performed between the three groups: A vs. C, ^+^ *p* < 0.05.

**Table 4:** Long-term clinical course of psychiatric patients with neural autoantibodies.

Parameter	Visit 1	Visit 2	Visit 3	Visit 4	Visit 5	Visit 6	Statistics Visit 1 vs. 4	Statistics Visit 1 vs. 6
Time course in months	0	3.4 ± 2.4	2.6 ± 1.7	5.4 ± 2.5	4.3 ± 2.4	4.6 ± 1.9	8.3 ± 3.1	17.0 ± 5.1
Presence of neural abs	32/32 (100%)	10/13 (77%)	3/4 (75%)	3/3 (100%)	4/4 (100%)	2/2 (100%)		
**Psychopathology**								
Disturbances of conciousness	0/32 (0%)	0/20 (0%)	0/15 (0%)	0/10 (0%)	0/7 (0%)	0/6 (0%)	1	1
Disturbances of orientation	0/32 (0%)	0/20 (0%)	0/15 (0%)	0/10 (0%)	0/7 (0%)	0/6 (0%)	1	1
Disturbances of attention and memory	24/32 (75%)	14/20 (70%)	10/15 (67%)	4/10 (40%)	4/7 (57%)	3/6 (50%)	0.059	0.328
Formal thought disorder	9/32 (28%)	7/20 (35%)	3/15 (20%)	2/10 (20%)	0/7 (0%)	0/6 (0%)	0.705	0.700
Worries and compulsions	0/32 (0%)	0/20 (0%)	0/15 (0%)	0/10 (0%)	0/7 (0%)	0/6 (0%)	1	1
Delusions	3/32 (9.4%)	2/20 (10%)	0/15 (0%)	2/10 (20%)	1/7 (14%)	0/6 (0%)	0.305	0.577
Disorders of perception	2/32 (6.2%)	0/20 (0%)	0/15 (0%)	0/10 (0%)	0/7 (0%)	0/6 (0%)	1	1
Ego disturbances	1/32 (3.1%)	0/20 (0%)	0/15 (0%)	0/10 (0%)	0/7 (0%)	0/6 (0%)	1	1
Disturbances of affect	11/32 (34%)	9/20 (45%)	6/15 (40%)	2/10 (20%)	4/7 (57%)	4/6 (67%)	0.465	0.188
Disorders of drive and psychomotor activity	8/32 (6.3%)	6/20 (30%)	0/15 (0%)	5/10 (50%)	3/7 (43%)	3/6 (50%)	0.238	0.328
Circadian disturbances	0/32 (0%)	0/20 (0%)	0/15 (0%)	0/10 (0%)	0/7 (0%)	0/6 (0%)	1	1
Other disturbances	4/32 (12.5%)	0/20 (0%)	0/15 (0%)	0/10 (0%)	0/7 (0%)	0/6 (0%)	0.556	1
**Therapy**								
Antipsychotic drugs	8/32 (25%)	7/20 (36%)	5/15 (33%)	6/10 (60%)	3/7 (43%)	2/6 (33%)	0.059	0.644
Antidepressive drugs	8/32 (25%)	6/20 (30%)	6/15 (40%)	5/10 (50%)	3/7 (43%)	3/6 (50%)	0.238	0.328
Antidementive drugs								
Immunotherapy	15/32 (47%)	3/20 (15%)	5/15 (33%)	4/10 (40%)	1/7 (14%)	0/6 (0%)	0.734	0.063
Steroids	11/32 (34%)	5/20 (25%)	5/15 (33%)	3/10 (30%)	1/7 (14%)	0/6 (0%)	1	0.153
IVIGs	2/32 (6.2%)	1/20 (5%)	0/15 (0%)	1/10 (10%)	0/7 (0%)	0/6 (0%)	1	1
Rituximab	2/32 (6.2%)	0/20 (0%)	0/15 (0%)	0/10 (0%)	0/7 (0%)	0/6 (0%)	1	1
**Affective Symptoms**								
Improvement	4/32 (12.5%)	5/20 (25%)	4/15 (27%)	2/10 (20%)	1/7 (14%)	0/6 (0%)	1	1
Deterioration	4/32 (12.5%)	3/20 (15%)	5/15 (33%)	4/10 (40%)	3/7 (43%)	3/6 (50%)	0.075	0.063
Stable	4/32 (12.5%)	2/20 (10%)	0/15 (0%)	0/10 (0%)	1/7 (14%)	1/6 (17%)	0.556	1
**Psychotic Symptoms**								
Improvement	8/32 (25%)	3/20 (15%)	2/15 (13%)	3/10 (30%)	0/7 (0%)	0/6 (0%)	1	0.309
Deterioration	0/32 (0%)	2/20 (10%)	1/15 (7%)	1/10 (10%)	1/7 (14%)	0/6 (0%)	0.238	1
Stable	1/32 (3.1%)	1/20 (5%)	0/15 (0%)	0/10 (0%)	0/7 (0%)	0/6 (0%)	0.238	1
**Cognitive Dysfunction**								
Improvement	5/32 (15.6%)	3/20 (15%)	3/15 (20%)	2/10 (20%)	0/7 (0%)	0/6 (0%)	0.664	0.570
Deterioration	11/32 (34%)	2/20 (10%)	2/15 (13%)	1/10 (10%)	0/7 (0%)	2/6 (33%)	0.233	1
Stable	4/32 (12.5%)	11/20 (55%)	4/15 (27%)	2/10 (20%)	2/7 (28%)	1/6 (17%)	0.616	1

Abbreviations: IVIG = intravenous immunoglobulins.

## Data Availability

Data are available on demand from the corresponding author.

## Supplementary Materials

The following supporting information can be downloaded at: https://www.mdpi.com/article/10.3390/antib12020034/s1, Table S1A: Suitability of patients for outpatient unit; Table S1B: Registration form for the outpatient clinic for autoantibody-mediated psychiatric disorders.
