# Supplementary material for: Long-Term Course of Neural Autoantibody-Associated Psychiatric Disorders: Retrospective Data from a Specifically Immunopsychiatric Outpatient Clinic
Source: Antibodies (Basel). 2023 May 8;12(2):34. doi: 10.3390/antib12020034 (PMC10204564; doi:10.3390/antib12020034)
Supplement: Supplementary file 1 [file antibodies-12-00034-s001.zip › antibodies-2249575-supplementary.pdf]

**Table S1A: Suitability of patients for outpatient unit**	
1	Patients with psychiatric symptoms and syndromes and the detection of autoantibodies in serum and/or cerebrospinal fluid
2	Patients with subacute onset of psychiatric symptoms and syndromes
3	Patients with psychiatric symptoms and syndromes and other autoimmune diseases, i.e., lupus erythematosus, Hashimoto's thyroiditis, Sjögren's syndrome, and autoantibody-associated vasculitis.
4	Patients with mainly psychiatric symptoms also suffering from seizures
5	Patients with mainly psychiatric symptoms also suffering from movement disorders
6	Patients with mainly psychiatric symptoms also suffering from disturbances of consciousness and/or cognition
7	Patients with mainly psychiatric symptoms also suffering from fever
8	Patients with mainly psychiatric symptoms also suffering from fluctuating psychiatric symptoms
9	Patients with mainly psychiatric symptoms also suffering from headaches
10	Patients with mainly psychiatric symptoms also suffering from recently discovered tumors
11	Patients with mainly psychiatric symptoms also suffering from neurological deficits
12	Patients with mainly psychiatric symptoms also suffering from speech and language disorders
13	Patients with mainly psychiatric symptoms also suffering from treatment resistance to standard psychopharmacological therapy
**Table S1B: Registration form for the outpatient clinic for autoantibody-mediated psychiatric disorders**	
Please answer these questions and bring this form with you to your first appointment with us:	
A. General	
1	Why do you wish to have us examine you?
2	What is your preliminary working diagnosis?
3	What is your name, and when were you born?
4	What is your telephone number and address?
B. Special questions	
1	Please describe your primary symptoms and/or problem.
2	Have you had any preliminary tests, if so, which, and with what results?
3	If available, please bring a written report and CD of your MRI exam (e.g., magnetic resonance imaging of your head).
4	Please bring a written report of your nerve fluid findings, if available.
5	Please bring, if available, a written report of your brainwave findings (electroencephalography)
6	What previous treatments have you undergone?
7	What medications have you taken in the past, what medications are you currently taking?
8	What illnesses do you have, previous diagnoses, etc.?
